# *Adhoc*: an R package to calculate *ad hoc* distance thresholds for DNA barcoding identification

**DOI:** 10.3897/zookeys.365.6034

**Published:** 2013-12-30

**Authors:** Gontran Sonet, Kurt Jordaens, Zoltán T. Nagy, Floris C. Breman, Marc De Meyer, Thierry Backeljau, Massimiliano Virgilio

**Affiliations:** 1 Royal Belgian Institute of Natural Sciences, OD Taxonomy and Phylogeny (JEMU), Vautierstraat 29, 1000 Brussels, Belgium; 2 Royal Museum for Central Africa, Department of Biology (JEMU), Leuvensesteenweg 13, 3080 Tervuren, Belgium; 3 University of Antwerp, Evolutionary Ecology Group, Groenenborgerlaan 171, 2020 Antwerp, Belgium

**Keywords:** Species identification, accuracy, precision, relative error, reference library, COI

## Abstract

Identification by DNA barcoding is more likely to be erroneous when it is based on a large distance between the query (the barcode sequence of the specimen to identify) and its best match in a reference barcode library. The number of such false positive identifications can be decreased by setting a distance threshold above which identification has to be rejected. To this end, we proposed recently to use an *ad hoc* distance threshold producing identifications with an estimated relative error probability that can be fixed by the user (e.g. 5%). Here we introduce two R functions that automate the calculation of *ad hoc* distance thresholds for reference libraries of DNA barcodes. The scripts of both functions, a user manual and an example file are available on the JEMU website (http://jemu.myspecies.info/computer-programs) as well as on the comprehensive R archive network (CRAN, http://cran.r-project.org).

## Introduction

**Figure 1.**
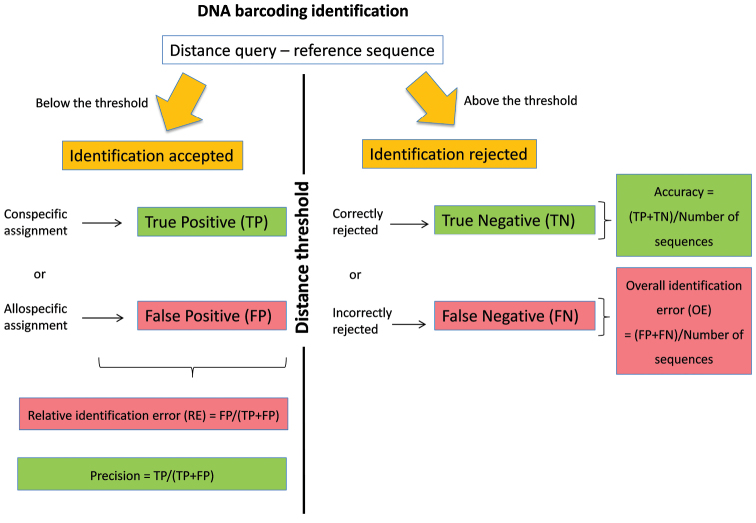
DNA barcoding identification using the best close match method.

The DNA barcoding initiative aims at providing a simple and standardised tool for specimen identification using a short DNA sequence from a specific region of the genome as a barcode (Hebert et al. 2003). The identification of a specimen using DNA barcoding is based on the comparison between its DNA barcode sequence (= query) and a reference library of DNA barcodes. These reference sequences satisfied a series of requirements that allow quality control (link to voucher specimen, trace files, and association with additional information such as primer and collection data). Among the approaches available for the assignment of a species name (Frézal and Leblois 2008, Austerlitz et al. 2009), methods based on sequence similarity are fast, easy and frequently applied as a first step to screen large reference libraries (Frézal and Leblois 2008). In this method, the species name of the reference sequence(s) showing the smallest genetic distance with the query (i.e. best match *sensu* Meier et al. 2006) is used for the identification (Ratnasingham and Hebert 2007). The identification provided by the best match method can be considered as true positive (TP) if a correct species name is assigned to the query or as false positive (FP) if an incorrect species name is assigned to the query (Figure 1). Yet, for many taxonomic groups, reference libraries are still incompletely representing the genetic diversity that can be found on specific and population levels. Some queries are therefore not represented by a conspecific DNA barcode in the library and will be erroneously identified according to the most similar allospecific reference barcode. Yet, the number of this sort of false positive identifications can be greatly reduced by assigning species names only when the distance between the query and its best DNA barcode match is below an arbitrary distance threshold value. With this best close match method (*sensu* Meier et al. 2006), identifications can still be TP or FP when the genetic distance between the query and its best match(es) is below the threshold. When this genetic distance is above the threshold (Figure 1), then either incorrect species name assignments can be correctly ignored (true negatives, TN) or correct species name assignments can be erroneously ignored (false negatives, FN). The determination of this distance threshold can be arbitrary (Ratnasingham and Hebert 2007) or can be based on the expected separation between intra- and interspecific distances (Meyer and Paulay 2005, Lefébure et al. 2006, Puillandre et al. 2012).

Recently, we proposed a general working strategy to deal with incomplete reference libraries of DNA barcodes (Virgilio et al. 2012). This method is based on *ad hoc* distance thresholds that are calculated for each library considering the estimated probability of relative identification errors. Indeed, by using each sequence of a reference library as a query against all other reference sequences, we can calculate (Virgilio et al. 2012, Figure 1) the relative identification error (RE) of the best close match method as FP/(TP+FP), its overall identification error (OE) as (FP+FN)/total number of queries, its accuracy as (TP+TN)/total number of sequences and its precision as TP/(TP+FP). The general procedure consists of 1) calculating the RE in a library of DNA barcodes for a number of arbitrarily chosen distance thresholds, 2) modelling the relation between distance thresholds and RE and 3) estimating the *ad hoc* threshold that would yield an estimated RE (e.g. 5%) for that particular library (Virgilio et al. 2012).

Here we introduce the R package "adhoc" including two functions, checkDNAbcd (“check DNA barcode”) and adhocTHR (“*ad hoc* threshold”), which automate this procedure and calculate the *ad hoc* distance threshold.

## Description of both functions

Both functions rely on the packages ape (Paradis et al. 2004), pegas (Paradis 2010) and spider (Brown et al. 2012). The first function, checkDNAbcd, imports a reference library of aligned DNA barcodes in FASTA format and provides basic descriptive statistics of the imported dataset, allowing a first quality check of the library. This function produces two tables containing species names, full sequence identifiers (as read by the function from the input file), and numbers of sequences and haplotypes for each species. CheckDNAbcd also returns the length of each reference sequence, calculates all pairwise distances and separates intra- and interspecific pairwise comparisons. The calculation of pairwise distances can be on the basis of simple uncorrected p-distances (representing the proportion of sites at which two sequences differ) or of several nucleotide substitution models such as the Kimura 2-parameter model (Kimura 1980), which is standardly used in DNA barcoding (Ratnasingham and Hebert 2007).

The second function, adhocTHR, utilises the output of the first function and performs best match and best close match identifications by taking each sequence of the reference library as a query against all other sequences of the library (Virgilio et al. 2012). For the best match identification, each query is identified as TP, FP or ambiguous false positive (FPambiguous, when both correct and incorrect species names are found as best matches). For the best close match identification, adhocTHR automatically evaluates each identification as TP, FP, FPambiguous, TN or FN and calculates the RE, OE, accuracy and precision at 30 arbitrary distance thresholds (equally distributed between zero and the largest distance observed between all pairs of query – best match). Relationships between distance thresholds and RE are then modelled through regression fitting. Regression is used to calculate the *ad hoc* distance threshold (Virgilio et al. 2012) producing an expected RE (5% by default). The function adhocTHR also produces a list of red-flagged matches (conspecific and allospecific matches responsible for the ambiguous identifications) and a table of red-flagged species names (species involved in the ambiguous identifications). The user has the possibility of modifying (1) the regression fitting (linear by default, or polynomial), (2) the number of arbitrary distance thresholds used for the fitting, (3) the estimated RE probability and (4) the treatment of ambiguous identifications. By default, the function treats ambiguous identifications as incorrect but they can optionally be ignored in the calculation or considered as correct. We recommend using this last option with caution since it will treat all red-flagged species involved in the same ambiguous identification as a single species.

As an indication, five minutes were necessary for each function to process a dataset of 5000 records (600-650 bp) on a personal computer (processor Intel Core i5 CPU M540, 2.53 GHz, 4 GB RAM with Windows 7 as operating system) using default parameters. Calculating the RE for more than 30 arbitrary distance thresholds is suggested to improve the fitting when computing time is not an issue.

When using reference libraries with particularly low levels of taxon coverage (Virgilio et al. 2010), reaching an estimated RE of 5% might not be possible, even at the most restrictive distance threshold (*viz.* distance threshold = 0.00) where only identical sequences are used for identification, all the other ones are discarded. In those cases the script will provide a warning message to inform the user that the script cannot find an *ad hoc* distance threshold for the chosen error probability.

This method has been developed for specimen identification. It is intended to optimise the identification success rate by adapting the distance threshold according to a RE estimated from a particular reference library. Hence, using this method for species delimitation requires a careful interpretation of the output (Collins and Cruickshank 2013). The estimation of the RE in DNA barcoding is an indispensable prerequisite, not only for forensic applications (Wells and Stevens 2008), but also for any further research relying on DNA barcoding identifications such as ecology or biodiversity inventories (Frézal and Leblois 2008).

**Figure 2.**
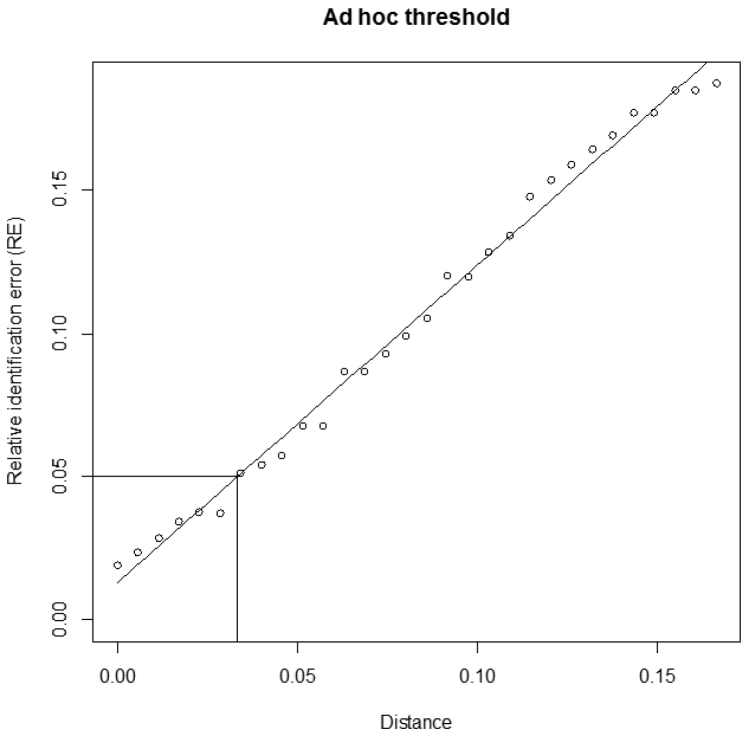
Estimation of the *ad hoc* distance threshold. Example of output obtained using the function adhocTHR with default settings (30 arbitrary distance thresholds, linear fit and an estimated relative identification error (RE) of 5%). The following message was given by the function: "for a RE of 0.05 use a threshold of 0.0334".

The script of both functions, a user manual and an example file are available on the JEMU website (http://jemu.myspecies.info/computer-programs) and on the comprehensive R archive network (CRAN, http://cran.r-project.org). The user manual suggests a few R commands to plot (1) the distribution of sequence lengths, (2) the distribution of intra- and interspecific pairwise distances and (3) a graph representing the RE obtained with the different arbitrary distance thresholds, the linear or polynomial fitting and the distance value corresponding to the *ad hoc* threshold (Figure 2).
